# Influence of mesenchymal stem cell-derived extracellular vesicles in vitro and their role in ageing

**DOI:** 10.1186/s13287-019-1534-0

**Published:** 2020-01-03

**Authors:** Juan Fafián-Labora, Miriam Morente-López, María José Sánchez-Dopico, Onno J. Arntz, Fons A. J. van de Loo, Javier De Toro, María C. Arufe

**Affiliations:** 1 Grupo de Terapia Celular y Medicina Regenerativa, Instituto de Investigación Biomédica de A Coruña (INIBIC), Complexo Hospitalario Universitario de A Coruña (CHUAC), 15006-A Coruña, Spain; 2 Departamento de Fisioterapia, Ciencias Biomédicas y Medicina, Facultad de Ciencias de la Salud, Universidad de A Coruña (UDC), As Xubias, 15006-A Coruña, Spain; 3 Experimental Rheumatology, Department of Rheumatology, Radboud University Medical Center, Nijmegen, 6525 GA The Netherlands

**Keywords:** Mesenchymal stem cell-derived extracellular vesicles, Ageing, Pluripotency, mTOR pathway

## Abstract

**Introduction:**

This study assessed whether mesenchymal stem cell (MSC)-derived extracellular vesicles influenced ageing and pluripotency markers in cell cultures where they are added.

**Methods:**

MSC-derived extracellular vesicles from old and young rat bone marrows were isolated by ultracentrifugation and were characterised by western blotting, nanoparticle tracking analysis (NTA) and transmission electron microscopy (TEM). They were added to young and old MSC cultures. Real-time quantitative reverse transcription polymerase chain reactions and western blot analysis were performed to check the markers of ageing (vinculin and lamin A), pluripotency markers (Nanog and Oct4) and components of the mTOR signalling pathway (Rictor, Raptor, AKT and mTOR) in these cell populations. Subsequently, microRNA (miR)-188-3p expression was transiently inhibited in young MSCs to demonstrate the influence of mTOR2 on MSC ageing.

**Results:**

Incubation with young MSC-derived extracellular vesicles decreased the levels of ageing markers and components of the mTOR pathway and increased the pluripotency markers from old MSC populations. By contrast, incubation of young MSCs with old MSC-derived extracellular vesicles generated the reverse effects. Inhibition of miR-188-3p expression in young MSCs produced extracellular vesicles that when incubated with old MSCs produced an increase in the levels of Rictor, as well as a decrease of phosphor-AKT, as indicated by a significant decrease in beta-galactosidase staining.

**Conclusions:**

MSC-derived extracellular vesicles affected the behaviour of MSC cultures, based on their composition, which could be modified in vitro. These experiments represented the basis for the development of new therapies against ageing-associated diseases using MSC-derived extracellular vesicles.

## Introduction

Cellular senescence, the result of complex phenotypic changes and ageing, constitutes a great risk factor that contributes to trigger different degenerative diseases. Unravelling the importance of the effect of ageing on mesenchymal stem cells (MSCs) is crucial in the development of new therapy based on these cells, focusing on the treatment of older people. The beneficial effects on damaged tissues are attributed to the paracrine activity of the MSCs, which include their soluble factors and extracellular vesicles (EVs). An interesting study indicated that MSC-derived EVs have significant potential as a novel alternative to whole-cell therapies in an experimental preclinical model of the inflammatory lung [1]. EVs transport mRNA, microRNA (miRNA) and proteins giving them the possibility of transporting biological information to the surrounding cells [2]. Previous results by our group indicated that proliferative, pluripotency and metabolism profiles of MSCs have significant differences influenced by the donor age [3]. We also demonstrated that immune profiles of MSC-derived EVs have age-dependent differences and they can be modified using miRNA [4]. The mammalian target of rapamycin (mTOR) is an amino acid sensor which perceives and incorporates different environmental signals. mTOR balances cell growth and nutrient availability and contributes to decisions on stem cell fate as a consequence of endogenous DNA damage [4]. mTOR also regulates cellular senescence and drives the infrastructure of bioenergetics [5]. Rapamycin inhibits the senescence effect produced by mTOR, which is mediating MSC proliferation potential through its effect on their self-renewal loss [6]. There are two signalling complexes, mTOR complex 1 (mTORC1) and mTOR complex 2 (mTORC2). mTORC2 plays an important role in endothelial senescence, evident by an increase in binding of Rictor, which is an essential component of mTORC2-directed phosphorylation of mTOR at Ser2481 and AKT (phosphor-AKT), producing an increase of β-galactosidase staining in senescence [7]. MicroRNA (miR)-188-3p has been shown to be a key regulator of age-associated bone loss in the bone marrow through its target on Rictor [8], and insights into the mechanism involved in MSC ageing suggest that interventions with miRNAs could modify the function of MSCs and their derived extracellular vesicles [4]. This study assessed whether EVs could modify MSC markers of ageing in vitro and whether Rictor has a key role through the mTORC2 pathway involving the ageing process.

## Material and methods

### Isolation and culture of MSCs

Animals were anesthetised with Fluorane (Izasa, A Coruña, Spain) and euthanised by the cervical dislocation method. The femoral bones were dissected from male Wistar rats (Animal Service, CHUAC, Spain) at different ages, young (14 days old) and old (270 days old). All methods were carried out following the approved guidelines of the Spanish Law (32/2007). All experimental protocols were approved by the Animal Ethical Committee of Galicia. The protocol published by Karaoz et al. [1] was used in this work. Briefly, the ends of the femoral bones were cut away, and a 21-gauge needle inserted into the shaft of the bone marrow was extruded by flushing with 4 ml D-Hank’s solution supplemented with 100 IU/ml penicillin-100 mg/ml streptomycin (Life Technologies, Madrid, Spain). The marrow plug suspension was dispersed by pipetting and filtered through a 70-μm mesh nylon filter (BD Biosciences, Bedford, MA, USA), successively centrifuged at 2000*g* for 10 min. The supernatant containing haematopoietic cells was discarded, and the cell pellet was resuspended in Roswell Park Memorial Institute (RPMI) medium supplemented with 10% (v/v) foetal bovine serum (FBS), 1% (v/v) penicillin and 1% (v/v) streptomycin (Life Technologies, Madrid, Spain). The MSCs were plated into 100-cm^2^ dish plates (Corning Inc., NY, USA) and incubated at 37 °C in a humidified atmosphere of 5% CO_2_. MSCs were isolated because of their ability to adhere to the culture plates. On the third day, red blood cells and other non-adherent cells were removed by a pre-plating technique, and fresh medium was added to allow further growth to the MSCs. The adherent MSCs grown to 70% confluence were defined as passage 0 (P0) cells. The culture medium was replaced every 3 or 4 days. MSCs were expanded for two passages before being used in the experiments. In 6-well plates (Corning Inc., NY, USA), 2.5 × 10^5^ MSCs from old individuals were cultured per well for 8 h, and 2 × 10^7^ particles of MSC-derived EVs from young individuals were added to these wells, and vice versa. MSCs were collected after 2, 3 and 6 days in culture with different MSC-derived EVs, and RNA and protein isolations were performed. Young MSCs were incubated with 40 nM miR-188-3p miRVAna™ inhibitor or 40 nM control negative miRVAna™ Mimic using the expression system and protocols of the manufacturer. Validation by reverse transcription polymerase chain reaction (RT-PCR) was done using TaqMan® MicroRNA Assays following the instructions of the manufacturer (Ambion, Applied Biosystems, Madrid, Spain). MSC cultures without added MSC-derived EVs were used as a control in all the experiments.

### Flow cytometry

To characterise the MSCs, they were washed twice in phosphate-buffered saline (PBS; Sigma-Aldrich, St. Louis, MO, USA) then pre-blocked with 2% rat serum in PBS. The following direct antibodies were used: phycoerythrin (PE)-conjugated mouse anti-human CD34 (1:20; DakoCytomation, Barcelona, Spain), fluorescein isothiocyanate (FITC)-conjugated mouse anti-rat CD45 (1:20; BD Pharmingen, Franklin Lakes, NJ, USA), PE-cyanine (Cy)5.5-conjugated mouse anti-rat CD90 (1:20; Immunostep, Salamanca, Spain) and allophycocyanin (APC)-conjugated mouse anti-rat CD29 (1:20; Immunostep, Salamanca, Spain). The cells were washed with PBS after 1 h of incubation with the corresponding antibody at room temperature. Fluorescence-activated cell sorting (FACS) data was generated by BD FACSDiva software (BD Science, San Jose, CA, USA). Negative control staining was performed using FITC-conjugated mouse IgG1K isotype, PE-conjugated mouse IgG1K isotype, PE-Cy5.5-conjugated mouse IgG1K isotype and APC-conjugated mouse IgG1K isotype (BD Pharmingen, Franklin Lakes, NJ, USA).

### Isolation of MSC-derived EVs

MSCs from young (14 days) and old (270 days) rats were cultured with RPMI 1640 medium with GlutaMAX™ supplement, 10% exosome-depleted FBS (Thermo Fisher Scientific, Waltham, MA, USA) and 100 IU/ml penicillin-100 mg/ml streptomycin (Life Technologies, Madrid, Spain). Cells were cultured to 80% confluence, and the supernatants were collected after 48 h. Supernatants were centrifuged at 2000×*g* for 10 min at 4 °C and filtered using a sterile 0.22-μm filter (GE Healthcare Life Sciences, Little Chalfont, UK) to eliminate debris, and they were transferred into new ultracentrifugation tubes (Beckman Coulter, Mississauga, Canada) and centrifuged at 100,000×*g* for 2 h at 4 °C in an Optimal-90K ultracentrifuge with a 60 Ti rotor (Beckman Coulter, Mississauga, Canada). The last supernatants containing exosome-depleted FBS were removed, and the pellets were resuspended in 200 μl PBS (MP Biomedicals, Illkrich-Graffenstaden, France).

### Nanoparticle tracking analysis of MSC-derived EVs

The Brownian motion of the particles in a NanoSight LM12 using Nanoparticle Tracking Analysis 2.3 software (NanoSight Ltd., Amesbury, UK) was used to calculate the EV size distribution after the ultracentrifugation. Total protein concentrations in MSC-derived EVs were determined with a Micro-bicinchoninic acid (BCA) kit (Thermo Fisher Scientific, Rockford, IL, USA), according to the manufacturer’s instructions.

### Electron microscopy

MSC-derived EVs were concentrated using Vivaspin concentrators (Sartorius, Gottingen, Germany). They were gathered up in small volumes of deionised water, which were then placed on nickel grids and allowed to dry for 45 min at 37 °C. The grids with MSC-derived EVs were fixed with 4% (w/v) paraformaldehyde (Sigma-Aldrich, St. Louis, MO, USA) for 10 min and then washed by dipping them onto several drops of deionised water. The grids were examined on a Jeol JEM1400 transmission electron microscope (Jeol Ltd., Tokyo, Japan).

### Fluorescence microscopy

MSCs (2.5 × 10^5^) from old and young individuals were cultured on slides (Sigma-Aldrich, St. Louis, MO, USA) pre-treated with poly-d-lysine (Sigma-Aldrich, St. Louis, MO, USA) in 6-well plates (Corning Inc., New York, NY, USA) for 8 h (day 1). MSC-derived EVs (2 × 10^7^) from young and old individuals were stained with 10 μM 3-3′-diethylthiacarbocyanineiodide (DiI) and added to each culture of MSCs; PBS (MP Biomedicals, Illkrich-Graffenstaden, France) was added as a control. At 2, 3 and 6 days, the cells were washed three times with PBS (MP Biomedicals, Illkrich-Graffenstaden, France) and fixed with 4% (w/v) paraformaldehyde (Sigma-Aldrich, St. Louis, MO, USA) for 10 min, and then the slides were mounted using ProLong® Gold antifade mountant with 4′,6-diamidino-2-phenylindole (DAPI; (Thermo Fisher Scientific, Carlsbad, CA, USA). The cells were examined with an Olympus BX61 microscope using a DP71 digital chamber (Olympus, Tokyo, Japan) with software DP-Controller and DP-Manager software. A NeoScope JCM-6000 Plus scanning electron microscope (Nikon-Izasa, Barcelona, Spain) was used to take supplementary images.

### Reverse transcription quantitative PCR analysis

Total RNA from culture cells was isolated with TRIzol® reagent (Thermo Fisher Scientific, Waltham, MA, USA). For miRNA detection, cDNA was generated from DNaseI-treated RNA, using a QuantiMir RT Kit (System Biosciences, Palo Alto, CA, USA), according to the manufacturer’s instructions. PCR products were amplified using specific primers for miRNAs, mmu-miR-188-3p and U6 small nuclear RNA (Thermo Fisher Scientific, Waltham, MA, USA). The amplification programme consisted of an initial denaturation at 50 °C for 2 min, followed by 95 °C for 10 min, and 50 cycles of annealing at 95 °C for 15 s and extension at 60 °C for 1 min. Primers for the amplification of rat genes are described in Table 1. The amplification programme consisted of an initial denaturation at 92 °C for 2 min, followed by 40 cycles of annealing at 95 °C for 15 s; annealing at 55–62 °C, depending on the gene, for 30 s; and extension at 72 °C for 15 s. PCRs were done in triplicate, with each set of assays repeated three times. To minimise the effects of unequal quantities of starting RNA and to eliminate potential sources of inconsistency, relative expression levels of each gene were normalised to ribosomal protein (HPTR) or U6 small nuclear RNA using the 2−ΔΔCT method x[9]. Control experiments utilised no reverse transcriptase.

**Table 1:** Specific primers for real-time reverse transcriptase polymerase chain reaction (RT-PCR) amplification, listed with their annealing temperature (AT)

Gene name	Fw primer	Rv primer	mRNA ID	AT (°C)
*Nanog*	atgcctcacacggagactgt	aagtgggttgtttgcctttg	NM_005103.4	61
*Oct4*	ctcctggagggccaggaatc	atatacacaggccgatgtgg	NM_00510	61
*Vinculin*	aggagaccttgcgaagacagg	gcggttgccacttgtttag	NM_001107248	61
*LMNA*	gagcaaagtgcgtgaggagt	tccccctccttcttggtatt	NM_001002016.2	61
*mTOR*	atcaagcaagcgacatctca	caggccttggttaccagaaa	NM_019906.1	61
*AKT*	gacgtagccattgtgaaggag	ccatcattcttgaggaggaagt	NM_033230.2	61
*Rictor*	gttggaaaaatggcacaagg	ctgtatgtagtgagggcttcgtt	NC_005101.4	61
*HPRT*	agccgaccggttctgtcat	agccgaccggttctgtca	NM_012583.2	61

*Fw* forward, *Rv* reverse

### Immunoblot analysis

Immunoblot analysis was performed with 40 μg total protein extracted from MSCs or 20 μg total protein extracted from MSC-derived EVs. Proteins were separated according to their molecular weight using sodium dodecyl sulphate-polyacrylamide gel electrophoresis (SDS-PAGE), with the percentage (w/v) bis-acrylamide (Sigma-Aldrich, St. Louis, USA) of the resolving gels being determined by the size of the proteins. Proteins were then transferred to nitrocellulose membranes using a semi-dry method, using buffer with 20% (v/v) methanol (Panreac, Barcelona, Spain) for small proteins (< 100 kDa) or 10% (v/v) methanol (Panreac, Barcelona, Spain) for large proteins (> 100 kDa). Nitrocellulose membranes were then incubated for 1 h with agitation at room temperature in blocking buffer, consisting of 5% (w/v) bovine serum albumin (BSA) for phospho-proteins and 5% (w/v) milk (Sigma-Aldrich, St. Louis, USA). The membranes were probed with antibodies diluted in blocking buffer at 4 °C overnight. The following day, the membranes were washed three times for 5 min with Tris-buffered saline with 0.1% (v/v) Tween® 20 (TBST). The membranes were then incubated for 1 h at room temperature in horseradish peroxidase (HRP)-conjugated secondary antibodies diluted in blocking buffer. Next, the membranes were washed three times in TBST buffer for 5 min with agitation and twice using Tris-buffered saline (TBS) for 5 min with agitation. An Amersham ECL Western Blotting Analysis System (GE Healthcare, Little Chalfont, UK) was used to visualise protein-binding antibodies. The blots were probed with antibodies directed against LMNA/C (Acrix); mTOR, Rictor, phosphor-mTOR, AKT and phosphor-AKT (Cell Signaling Technology, Beverly, MA, USA); Tsg101, calnexin and CD63 (Abcam, Cambridge, MA, USA); and β-actin (Sigma-Aldrich, St. Louis, MO, USA). Adequate concentrations for each antibody were determined empirically. Blot images were digitised using a LAS 3000 image analyser (GE Healthcare, Little Chalfont, UK). Densitometry analysis of band intensities was performed using ImageQuant 5.2 software (GE Healthcare, Little Chalfont, UK).

### Senescence-associated beta-galactosidase staining

Cytochemical staining of senescence-associated beta-galactosidase (SA-β-gal) with 5-bromo-4-chloro-3-indolyl-β-d-galactopyranoside (X-Gal) was performed as previously described [7]. Cells were fixed and then stained with freshly prepared SA-β-gal staining solution overnight at 37 °C, according to the manufacturer’s protocols (Cell Signaling Technology, Beverly, MA, USA). The experiments were conducted three times, with ten images being acquired of each treatment using an Olympus BX61 microscope (Olympus, Tokyo, Japan). Images were quantified with ImageJ software, with the intensity of SA-β-gal staining being expressed as arbitrary units.

### Statistical analysis

All experiments were performed in triplicate, and one representative was shown in this paper. Non-parametric statistical analysis was performed by Mann-Whitney *U* and Kruskal-Wallis tests using GraphPad Prism 6 (GraphPad Software, La Jolla, USA). *P* values < 0.05 were considered statistically significant. All the results were presented as standard errors of the mean.

## Results

**Fig. 1:**
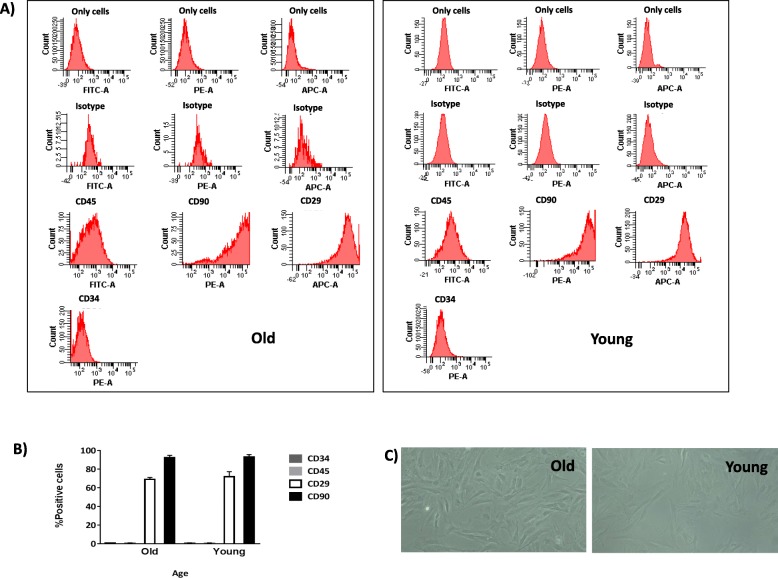
Characterisation of MSCs from the rat bone marrow at several ages. **a** One representative fluorescence-activated cell sorting (FACS) assay is shown for old MSCs and young MSCs. The antibody is indicated at the top of each plot and its linked fluorochrome at the bottom. **b** The plot shows the percentage of positive MSC markers (CD29 and CD90) and negative haematopoietic markers (CD34 and CD45). **c** Representative images (magnification, × 20) of old and young MSCs

Characterisation of both young and old populations of MSCs by flow cytometry revealed no statistically significant differences in levels of mesenchymal and haematopoietic markers (Fig. 1a). Cells positive for CD45 and CD34 represented less than 1% of cells positive for CD29 (70 ± 5%) and cells positive for CD90 (90 ± 5%), in all groups studied (Fig. 1b). There were no morphological differences between young and old MSCs (Fig. 1c), with both of them presenting as spindle-shaped.

**Fig. 2:**
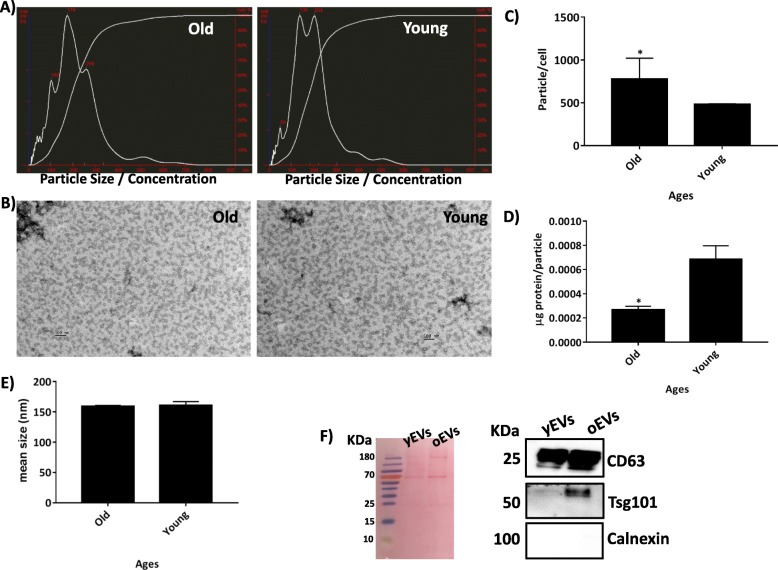
NTA study of MSC-derived EVs at several ages. **a** Representative results from the NTA assay of old and young MSC-derived EVs. **b** Electron micrographs of MSC-derived EVs from the old and young groups of rats (scale bar = 100 nm). **c** The number of particles per cell at different age groups by NTA assay. **d** Concentration of protein per particle at different age groups as determined by NTA assay. **e** Mean size of particles, expressed in nanometres, for different age groups as determined by NTA assay. *P < 0.05 compared with the former group was considered statistically significant using Mann-Whitney *U* and Kruskal-Wallis tests. **f** Ponceau S staining for protein content for 20 μg MSC-derived EVs and their immunoblot staining for exosome markers CD63, TSG101 and Calnexin

Representative graphics obtained by nanoparticle tracking analysis (NTA) from old and young MSD-derived EVs are shown in Fig. 2a. MSC-derived EVs were visualised by electronic microscopy as 70–80-nm-diameter vesicles in both old and young populations (Fig. 2b). The production of MSC-derived EVs increased with increasing donor age in a statistically significant way (52%; Fig. 2c). By contrast, the protein to particle ratio of MSC-derived EVs decreased with increasing donor age in a statistically significant way (37.5%; (Fig. 2d). The diameter of EVs as determined by NTA was 160 ± 18 nm and was not statistically significantly different between the young and old groups (Fig. 2e). Western blotting analysis indicated that the MSC-derived EVs contained exosome-associated proteins CD63 and TSG101 but not calnexin (Fig. 2f).

**Fig. 3:**
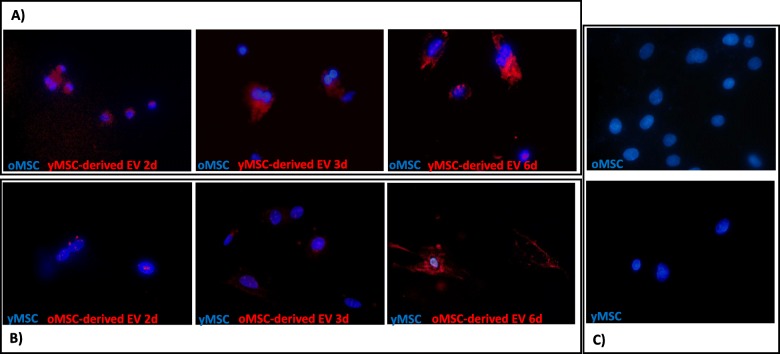
Internalisation of MSC-derived EVs as observed by fluorescence microscopy. Images from fluorescence microscopy (magnification, × 40) of the nuclei of MSCs stained with 4′,6-diamidino-2-phenylindole (DAPI) and MSC-derived EVs stained with DiI. **a** Old MSCs cultured with young MSC-derived EVs at 2, 3 and 6 days. **b** Young MSCs cultured with old MSC-derived EVs at 2, 3 and 6 days. **c** Control of young MSCs and old MSCs without added EVs. One representative experiment is shown. oMSC, MSCs from the old group; yMSC, MSCs from the young group; oEV, MSC-derived EVs from the old group; yEV, MSC-derived EVs from the young group

Internalising of old MSC-derived EVs into young MSCs was observed after 2 days in culture, and there was an increase in EVs inside the MSCs with time (Fig. 3a). A similar effect was observed by fluorescence microscopy in old MSCs co-cultured with young MSC-derived EVs (Fig. 3b). Additional file 1: Figure S1 shows an old MSC with its cytoplasm full of young MSC-derived EVs, at a magnification of × 100.

**Fig. 4:**
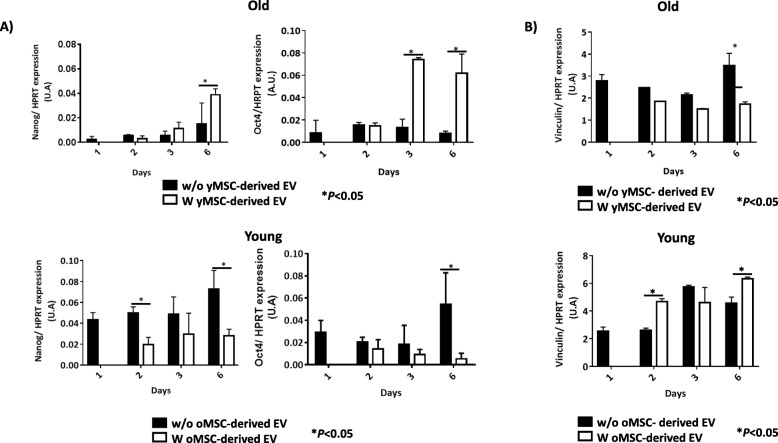
Study of the self-renewal potential in MSCs. **a** Histograms represent *Nanog* and *Oct4* gene expression normalised with *HPRT*, a housekeeping gene, in old MSCs with or without young MSC-derived EVs (upper). Histograms represent *Nanog* and *Oct4* gene expression normalised with *HPRT*, a housekeeping gene, in young MSCs with or without old MSC-derived EVs (bottom). **b** The histograms represent the gene expression of *vinculin* normalised by *HPRT*, a housekeeping gene, in old MSCs with or without young MSC-derived EVs (upper). The histograms represent the gene expression of *vinculin* gene expression normalised by *HPRT*, a housekeeping gene, in young MSCs with or without old MSC-derived EVs (bottom). *P < 0.05 compared with the former group was considered statistically significant using Mann-Whitney *U* and Kruskal-Wallis tests. Control, MSCs cultured with growth medium without EVs; oMSC, MSCs from the old group; yMSC, MSCs from the young group; oEV, MSC-derived EVs from the old group; yEV, MSC-derived EVs from the young group

Genes *Nanog* and *Oct4*, which are pluripotency markers in MSCs, showed statistically significant increased expression in old MSCs treated with young MSC-derived EVs at different times of incubation, compared to untreated cells (*P <* 0.05; Fig. 4a). By contrast, young MSCs treated with old MSC-derived EVs showed statistically significant lower *Nanog* and *Oct4* gene expression after 2 days of incubation (*P* < 0.05; Fig. 4a). *Vinculin*, a senescence marker, had a statistically significant decrease in its expression at 6 days for old MSCs treated with young MSC-derived EVs (*P* < 0.05; Fig. 4b), while it was increased statistically in young MSCs treated with old MSC-derived EVs (*P* < 0.05; Fig. 4b).

**Fig. 5:**
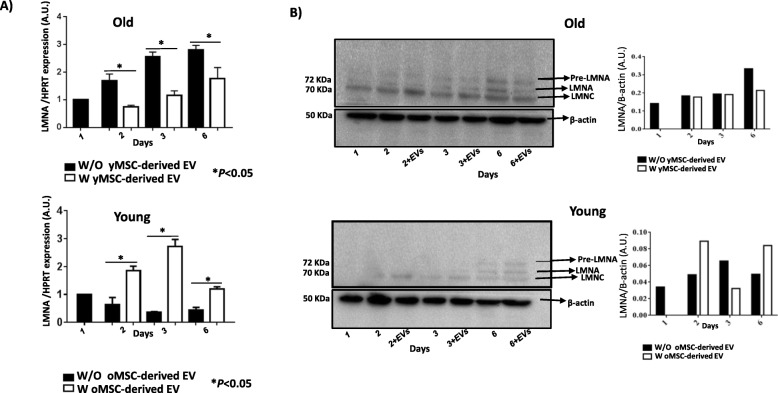
Study of senescence in MSCs. **a** The histograms represent the gene expression of *LMNA* normalised by *HPRT*, a housekeeping gene, in old and young MSCs with or without young or old MSC-derived EVs. *P < 0.05 compared with the control was considered statistically significant using Mann-Whitney *U* and Kruskal-Wallis tests. Control, MSCs cultured with growth medium without EVs; oMSC, MSCs from the old group; yMSC, MSCs from the young group; oEV, MSC-derived EVs from the old group; yEV, MSC-derived EVs from the young group. **b** Western blot analysis of pre-lamin A and their isoforms, lamin A/C, from old and young MSCs with or without old or young MSC-derived EVs at different times (2, 3 and 6 days) and LMNA densitometry analysis normalised with respect to β-actin, using Image Quant 5.2. The molecular weight of each protein is shown on the left. One representative experiment is shown. The gels have been run under the same experimental conditions

Gene *LMNA* and its isoforms, as well as *vinculin*, which were senescence markers in MSCs, showed a statistically significant decrease in expression in old MSCs treated with young MSC-derived EVs at different times of incubation, compared to untreated cells (*P* < 0.05; Fig. 5a). Young MSCs treated with old MSC-derived EVs also presented with statistically higher *LMNA* gene expression after 2, 3 and 6 days of incubation (*P* < 0.05; Fig. 5a). Validation of levels of LMNA and its isoforms at the protein level indicated that old MSCs treated with young MSC-derived EVs had a decrease in the expression of isoforms lamin A/C at 6 days (Fig. 5b), while young MSCs treated with old MSC-derived EVs showed an increased expression at 6 days (Fig. 5b).

**Fig. 6:**
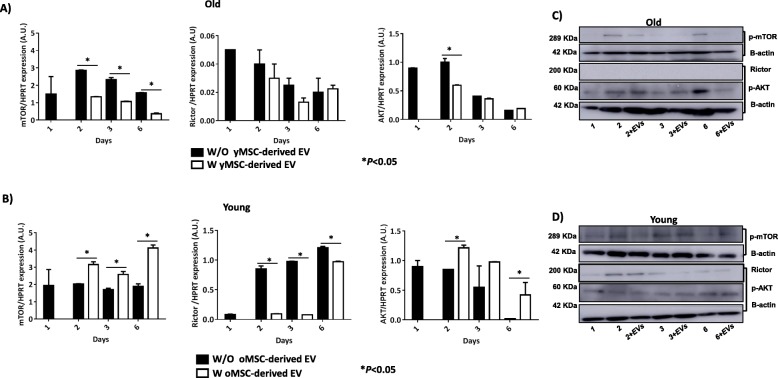
Study of mTOR family in MSCs. **a** The histograms represent the gene expression of *mTOR*, *Rictor* and *AKT* normalised by *HPRT*, a housekeeping gene, in old MSCs with or without young MSC-derived EVs at different times (2, 3 and 6 days). **b** The histograms represent the gene expression of *mTOR*, *Rictor* and *AKT* gene expression normalised by HPRT, a housekeeping gene, in young MSCs with or without old MSC-derived EVs at different times (2, 3 and 6 days). *P < 0.05 compared with the former group was considered statistically significant using Mann-Whitney *U* and Kruskal-Wallis tests. **c** Western blot analysis of phospho-mTOR, Rictor and phospho-AKT from old MSCs with or without young MSC-derived EVs at different times (2, 3 and 6 days). **d** Western blot analysis of phospho-mTOR, Rictor and phospho-AKT from young MSCs with or without old MSC-derived EVs at different times (2, 3 and 6 days). The molecular weight of each protein is shown on the left. One representative experiment is shown. The gels have been run under the same experimental conditions. Control, MSCs cultured with growth medium without EVs; oMSC, MSCs from the old group; yMSC, MSCs from the young group; oEV, MSC-derived EVs from the old group; yEV, MSC-derived EVs from the young group

Study of the members of the mTOR pathway revealed statistically significant decreased expression of *mTOR* (*P* < 0.05) in old MSCs treated with young MSC-derived EVs, after 2 and 6 days (*P* < 0.05; Fig. 6a). *Rictor* and *AKT* expression was also decreased; however, it was not statistically significant (Fig. 6a). By contrast, in young MSCs treated with old MSC-derived EVs, *mTOR* and *AKT* gene expression was significantly higher (*P* < 0.05) after 2, 3 and 6 days. *Rictor* revealed a statistically significant decrease in expression when young MSCs were treated with old MSC-derived EVs, after 2, 3 and 6 days (*P* < 0.05; Fig. 6b). Western blot analysis indicated that levels of phosphor-mTOR and phosphor-AKT were decreased in old MSCs treated with young MSC-derived EVs, while Rictor was not detected (Fig. 6c). By contrast, western blot analysis indicated that phosphor-mTOR was increased in young MSCs treated with old MSC-derived EVs. In addition, Rictor was detected in young MSCs treated with old MSC-derived EVs after 2 days in culture (Fig. 6d).

**Fig. 7:**
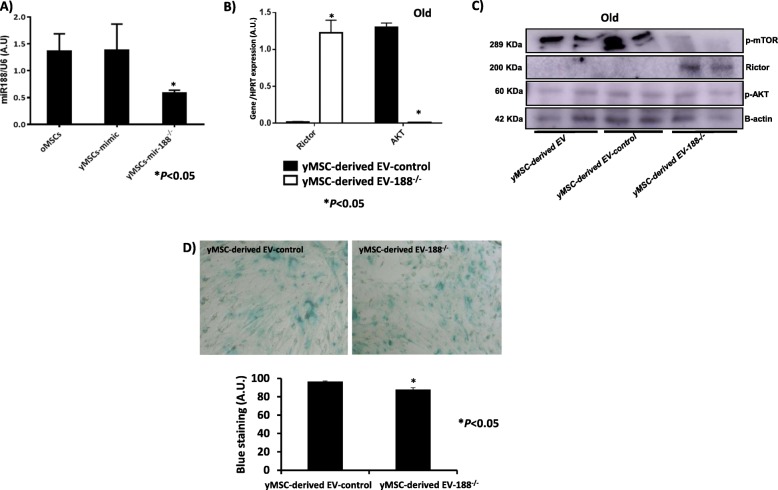
Effect of inhibition of miR-188-3p in MSCs. **a** miR-188-3p expression in old MSCs and young MSCs transfected with mimic or miR-188-3p inhibitor, as determined by RT-qPCR analysis normalised with the expression of U6 small nuclear RNA. **b** *Rictor* and *AKT* gene expressions using RT-qPCR analysis normalised with the expression of *HPRT* in old MSCs treated with young MSC-derived EVs mimic used as a control and young MSC-derived EVs miR188^−/−^. *P < 0.05 compared with control was considered statistically significant using Mann-Whitney *U* and Kruskal-Wallis tests. **c** Rictor and phosphor-AKT levels in old MSCs treated with young MSC-derived EV mimic used as control and young MSC-derived EVs miR188^−/−^, using western blot analysis normalised by the expression of β-actin. **d** Representative images (magnification, × 20) of senescence-associated beta-galactosidase (SA-β-gal) staining of old MSCs with young MSC-derived EVs and with young MSC-derived EVs modified with miR-188-3p inhibitor added to the culture media, showing a higher number of negative cells in young MSC-derived EVs miR-188^−/−^-treated cells in the densitometry analysis of the staining signal (bottom). Control, MSCs cultured with growth medium without EVs

A statistically significant decrease in the miR-188 expression was obtained in young MSCs when treated with the inhibitor of miR-188, compared with mimic-miR-188 used as a control to confirm transfection efficiency (*P* < 0.05; Fig. 7a). Young MSC-derived EVs-188^−/−^ were used to treat old MSCs, resulting in a statistically significant increase in the *Rictor* expression in this population, compared with old MSCs treated with only young MSC-derived EVs (Fig. 7b). By contrast, the gene *AKT* showed decreased expression. These results were independently validated by western blot (Fig. 7c), where old MSCs treated with young MSC-derived EVs-188^−/−^ produced an increased level of Rictor and a decreased level of phosphor-AKT. Young MSC-derived EVs-188^−/−^ produced a statistically significant decrease in the number of cells staining positively for SA-β-gal (Fig. 7d).

## Discussion

The current study indicated that MSC-derived EVs might be involved in the senescence process, which could be influenced by Rictor through mTORC2. MSCs have been used therapeutically in regenerative medicine for their differentiation and secretory potential. Biancone et al. [2] have reported that MSCs can modulate gene expression by releasing extracellular vesicles to maintain physiological processes.

MSCs from two different age groups of rats were characterised by flow cytometry (Fig. 1), and our results were consistent with those of Jin et al. [10]. All the cells had the ability to adhere to the plastic culture dish, which was an inherent characteristic of MSCs, and the groups were able to differentiate towards several mesoderm lineages, results that were similar to a previously published report [11]. The size of EVs was determined by NTA, which calculated the size from the total concentration of vesicles in solution, following the technique used by Gercel-Taylor et al. [12] and validated by electron microscopy (Fig. 2b). We found an increase in the production of MSC-derived EVs from the old group of rats compared to the young group (Fig. 2c). A rational explanation for this fact could be as a compensation mechanism by old MSCs because of a decrease in their protein to particle ratio (Fig. 2d). These results were similar to those previously published by our group [4]. In addition, the MSC-derived EVs were positives for CD63 and Tsg101 but negative for calnexin, by western blot (Fig. 2f), meeting the requirements for their definition as EVs as reported by the International Society for Extracellular Vesicles (ISEV) [13].

There is an interesting study about the mechanisms of EV uptake by target cells, such as endocytosis mediated by clathrin or caveolin-dependent, macropinocytosis and phagocytosis [5], mechanisms involving cell surface membrane fusion with lipidic participation [6]. In addition, by fluorescence microscopy, this current study demonstrated the internalisation of MSC-derived EVs labelled with DiI, a process that was independent of the age of the donor (Fig. 3). These results were similar to other studies where dendritic cells [8] and melanoma cells [14] were used. In addition, some DiI label was observed in the extracellular region that could represent lipids from the membranes of MSC-derived EVs, as their lipid composition was abundant in complex phosphoryl derivatives of sphingosine and choline, which would support the plasma membrane renewing [15]. Young MSC-derived EVs labelled with DiI inside old MSCs is shown in Additional file 1: Figure S1.

The discovery of epigenetic mechanisms has placed the dynamic of global chromatin centrally to the following of pluripotency, and lineage progression of human embryonic stem cells because of the histone deacetylation is necessary to express *Oct4* and *Nanog* in these cells [16]. Therefore, we performed genetic studies to evaluate the expression of those pluripotency markers, which are important transcription factors in controlling the expression of multiples genes associated with MSC pluripotency pathways. Reduced expression of these genes due to inactivation in in vitro culture under normoxia [11] results in reduced proliferation and pluripotency capacities [17]. It was observed that pluripotency expression markers changed when MSCs were incubated with MSC-derived EVs. Old MSCs showed increased expression of *Nanog* and *Oct4* when they were cultured with young MSC-derived EV. By contrast, young MSCs cultured with old MSC-derived EV showed decreased expression of these transcription factors (Fig. 4a). As such, we confirmed that EV influenced the self-renewal capacity of MSCs at the genetic level, consistent with Jo et al. [18], who used a system of nanovesicles engineered from embryonic stem cells. Merino-González et al. [19] reported that the secretion of soluble factors and the release of extracellular vesicles, such as exosomes, could mediate cellular communication, inducing cell differentiation and self-renewal.

*Vinculin* gene expression was assessed in this current study because it was involved in contractility and cellular adhesion [20]. Vinculin is a well-characterised F-actin binding protein localised in focal adhesions as well as in cell-adherence junctions and undergoes changes in the expression both at the proteomic and genetic levels in relationship with age. A decrease in the expression of this gene was observed in old MSC cultured with young MSC-derived EVs, while an increase was found in young MSCs cultured with old MSC-derived EVs (Fig. 4b). Another ageing marker studied was *LMNA*, whose expression is increased in premature ageing conditions, as is prelamin A [21]. An increase in the expression of the three lamin isoforms was observed by western blot technique in young MSCs co-cultured with old MSC-derived EVs, while old MSCs cultured with young MSC-derived EVs showed decreased levels (Fig. 5a, b), with the highest levels being found after 6 days. All these results indicated that EVs contained factors which either stimulated or inhibited targets in the cells to which they were added, consistent with the findings of Fierabracci et al. [22]. As such, we evaluated the changes at the protein levels and confirmed the influence of MSC-derived EVs on senescence of MSCs in vitro. This discovery suggested that old MSC-derived EVs contained “age-promoting” factors [23], which may be responsible for the age-associated decline in stem cells, self-renewal and pluripotency, which are influenced by ageing. It was an important concept in the understanding of the ageing process and in developing EV-based therapies.

Therefore, mTOR pathway represents a potential therapeutic target for improving defective, aged stem cells [4, 24]. *mTOR* showed decreased expression at the transcriptional (Fig. 6a) and post-transcriptional (phosphor-mTOR; Fig. 6c) levels when young MSC-derived EVs were incubated with old MSCs. By contrast, we found the opposite results at the transcriptional (Fig. 6b) and post-transcriptional (Fig. 6d) levels when old MSC-derived EVs were incubated in young MSC cultures. Rictor did not show any variation in old MSCs treated with young MSC-derived EVs at the transcriptional level, and it was not detected at the post-transcriptional level (Fig. 6a, c). It could be able because the expression of Rictor in young MSCs is very low, and it is corroborated at post-transcriptional level (Fig. 6c). Young MSC-derived EVs produced a reduction in *AKT* gene and AKT expressions (Fig. 6a, c). We observed that young MSC-derived EVs were more effective than old MSC-derived EVs in their influence on MSCs in vitro, as reported by several authors [25], except in the case of Rictor expression. The results obtained at the transcriptional and post-transcriptional levels showed a similar trend, although delayed in time, and this could be because unique features of MSCs are controlled by a dynamic interplay between extrinsic signalling pathways and regulation by epigenetic events not studied in this model. MiR-188-3p directly targets *Rictor*, as demonstrated by Li et al. [26] and Gharibi et al. [27], preventing the development of an age-related phenotype and maintaining MSC morphology of early passage cells, with high clonogenic frequency and enhanced proliferative capacity. Young MSCs showed knockdown on treatment with the antagonist of miR-188-3p (Fig. 7a) where miR-188-3p levels of the expression were significantly decreased, as well as its expression level in transfected young MSC-derived EVs (data not shown). These results were consistent with those published by Zou et al. [28] where miR-188-3p showed the same expression pattern in the cells as well as into their exosomes. Young MSC-derived EVs miR-188^−/−^ increased the expression of *Rictor* and decreased that of *AKT* (Fig. 7b) at the transcriptional level, and phosphorylated forms of mTOR and AKT were confirmed by western blotting (Fig. 7c), accomplished with a significant reduction of SA-β-gal staining-positive cells in old MSCs treated with young MSC-derived EVs miR-188^−/−^ versus old MSCs treated only with regular young-derived EVs (Fig. 7d). We produced an inhibition of phosphor-AKT in old MSCs through the increase of *Rictor* expression, by inhibition of miR-188-3p, which induced rejuvenation of old MSCs, as evident by a reduction of their beta-galactosidase staining. Therefore, bio-products from MSCs, such as extracellular vesicles, represent a promising new way of reprogramming resident cells in several diseases, such as acute and chronic kidney disease [29], or facilitating wound healing by promoting collagen synthesis and angiogenesis [30].

In conclusion, extracellular vesicles are a promising new approach to reprogramming cellular lines, modifying the expression of target families like mTOR involved in ageing, through their miR cargo.

## Supplementary information

**Additional file 1:** **Figure S1.** Young MSCs-derived EVs labelled with DiI inside old MSCs is shown.

## Data Availability

All data generated during this study are included in this published article and its supplementary information files.

## Abbreviations

DAPI: 4′,6-Diamidino-2-phenylindole
DiI: 3-3′-Diethylthiacarbocyanineiodide
EVs: Extracellular vesicles
HPRT: Hypoxanthine-guanine phosphoribosyltransferase
miRNA: MicroRNA
MSCs: Mesenchymal stem cells
MSC-derived EVs: Mesenchymal stem cell-derived extracellular vesicles
NTA: Nanoparticle tracking analysis
oEV: Mesenchymal stem cell-derived extracellular vesicles from the old group
oMSC: Mesenchymal stem cells from the old group
qRT-PCR: Real-time quantitative reverse transcription polymerase chain reaction
SA-β-gal: Senescence-associated beta-galactosidase
yEV: Mesenchymal stem cell-derived extracellular vesicles from the young group
yMSC: Mesenchymal stem cells from the young group
