# Supplementary figures and images for: Influence of mesenchymal stem cell-derived extracellular vesicles in vitro and their role in ageing
Source: Stem Cell Res Ther. 2020 Jan 3;11:13. doi: 10.1186/s13287-019-1534-0 (PMC6942375; doi:10.1186/s13287-019-1534-0)

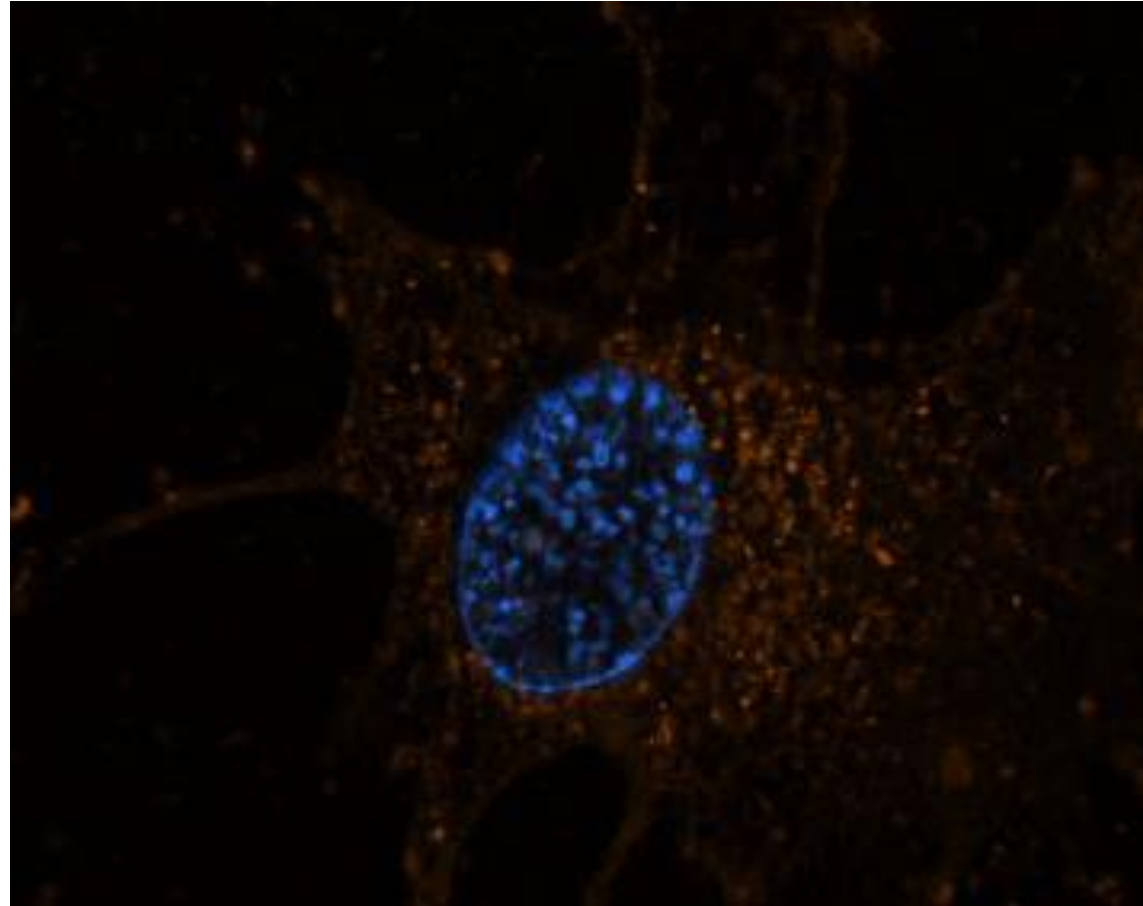

Supplement: Supplementary file 1 — Additional file 1: Figure S1. Young MSCs-derived EVs labelled with DiI inside old MSCs is shown. [file 13287_2019_1534_MOESM1_ESM.pdf]
